# Intermittent Hypoxic Conditioning Rescues Cognition and Mitochondrial Bioenergetic Profile in the Triple Transgenic Mouse Model of Alzheimer’s Disease

**DOI:** 10.3390/ijms22010461

**Published:** 2021-01-05

**Authors:** Sónia C. Correia, Nuno J. Machado, Marco G. Alves, Pedro F. Oliveira, Paula I. Moreira

**Affiliations:** 1 Center for Neuroscience and Cell Biology (CNC), University of Coimbra, 3004-504 Coimbra, Portugal; nihonmeru@yahoo.co.uk; 2 Center for Innovative Biomedicine and Biotechnology (CIBB), University of Coimbra, 3004-504 Coimbra, Portugal; 3 Institute for Interdisciplinary Research, University of Coimbra, 3030-789 Coimbra, Portugal; 4 Unit for Multidisciplinary Research in Biomedicine (UMIB), Department of Anatomy, Institute of Biomedical Sciences Abel Salazar (ICBAS), University of Porto, 4050-313 Porto, Portugal; alvesmarc@gmail.com; 5 QOPNA & LAQV, Department of Chemistry, University of Aveiro, 3810-193 Aveiro, Portugal; p.foliveira@ua.pt; 6 Laboratory of Physiology, Faculty of Medicine, University of Coimbra, 3000-548 Coimbra, Portugal

**Keywords:** Alzheimer’s disease, brain cortex, cognition, intermittent hypoxic conditioning, mitochondrial bioenergetics and dynamics, synaptic integrity, 3×Tg-AD mouse model

## Abstract

The lack of effective disease-modifying therapeutics to tackle Alzheimer’s disease (AD) is unsettling considering the actual prevalence of this devastating neurodegenerative disorder worldwide. Intermittent hypoxic conditioning (IHC) is a powerful non-pharmacological procedure known to enhance brain resilience. In this context, the aim of the present study was to investigate the potential long-term protective impact of IHC against AD-related phenotype, putting a special focus on cognition and mitochondrial bioenergetics and dynamics. For this purpose, six-month-old male triple transgenic AD mice (3×Tg-AD) were submitted to an IHC protocol for two weeks and the behavioral assessment was performed at 8.5 months of age, while the sacrifice of mice occurred at nine months of age and their brains were removed for the remaining analyses. Interestingly, IHC was able to prevent anxiety-like behavior and memory and learning deficits and significantly reduced brain cortical levels of amyloid-β (Aβ) in 3×Tg-AD mice. Concerning brain energy metabolism, IHC caused a significant increase in brain cortical levels of glucose and a robust improvement of the mitochondrial bioenergetic profile in 3×Tg-AD mice, as mirrored by the significant increase in mitochondrial membrane potential (ΔΨm) and respiratory control ratio (RCR). Notably, the improvement of mitochondrial bioenergetics seems to result from an adaptative coordination of the distinct but intertwined aspects of the mitochondrial quality control axis. Particularly, our results indicate that IHC favors mitochondrial fusion and promotes mitochondrial biogenesis and transport and mitophagy in the brain cortex of 3×Tg-AD mice. Lastly, IHC also induced a marked reduction in synaptosomal-associated protein 25 kDa (SNAP-25) levels and a significant increase in both glutamate and GABA levels in the brain cortex of 3×Tg-AD mice, suggesting a remodeling of the synaptic microenvironment. Overall, these results demonstrate the effectiveness of the IHC paradigm in forestalling the AD-related phenotype in the 3×Tg-AD mouse model, offering new insights to AD therapy and forcing a rethink concerning the potential value of non-pharmacological interventions in clinical practice.

## 1. Introduction

With a worldwide population that is aging at a rapid pace, the prevalence of AD is now reaching alarming proportions emerging as one of the major global epidemics of the 21st century [1]. Bereft of a cure, AD is currently the most common type of dementia, affecting more than 44 million people worldwide [2,3,4], this number being projected to triplicate by 2050. Within this scenario, AD represents a major frontier in medical research, a major challenge for the pharmaceutical industry, and a burden for society.

From a clinical perspective, AD has been conceptualized as a continuum that begins with a long asymptomatic or preclinical phase that typically progresses to dementia [5]. The clinical diagnosis of AD represents the end-stage of a decade-long silent disease process since the disruption of brain structure and function precede the discernible clinical manifestation of the disease by 20–30 years [6]. The cardinal symptoms of AD are an initial impairment in episodic memory that progresses continuously to a severe cognitive decline, and behavioral disturbances [7,8]. Neuropathologically, the AD signature is defined by the presence of extracellular deposition of Aβ peptide in senile plaques and the intracellular accumulation of neurofibrillary tangles containing hyperphosphorylated tau protein [9]. Despite the limited knowledge regarding the molecular roots behind AD, mitochondrial abnormalities have also been pinpointed as one of the earliest and strongest events during the pathological course of the disease [10].

More than 100 years after the first case report and the groundbreaking advances in understanding AD pathophysiology, the management of this complex disorder only provides symptomatic relief with no effective disease-modifying therapeutics able to halt the neurodegenerative processes before reaching a “point of no return”. As a matter of fact, according to the Pharmaceutical Research and Manufacturers of America (PhRMA), between 1998 and 2017, 146 anti-AD drugs failed in clinical practice worldwide, and only four have been marketed successfully. In this sense, the successive failure of pharmacotherapy encourages the “hunt” for new alternative interventions directed to AD.

Over the last decades a growing interest on the potential therapeutic application of conditioning in the context of brain disorders has emerged [11,12,13]. Conditioning is an adaptive biological process activated through the exposure to a sub-threshold insult (e.g., hypoxia, physical exercise, temperature) able to confer resistance to a deleterious brain event [14,15]. In 2009, it was reported for the first time that IHC counteracts AD-related cognitive decline and brain damage in a rodent model [16]. However, to date the mechanisms underlying IHC-mediated brain tolerance against AD phenotype remain elusive. Taking into account that mitochondria have been posited at the hub of conditioning phenomenon [17,18,19], the present study was undertaken to investigate if IHC is able to confer a long-lasting protection against AD-related phenotype in the 3×Tg-AD mouse model and to unveil the potential mitoprotective adaptations. 3×Tg-AD has been described as a progressive model of familial AD that develops subtle memory deficits at four months of age, Aβ pathology around 6 months of age and tau pathology after 12 months of age [20]. Herein, six-month-old 3×Tg-AD mice were submitted to an IHC protocol for a two-week period and mice were sacrificed at nine months of age. In the brain cortex, the following parameters were evaluated: (i) neuropathological hallmarks (Aβ and p-tau); (ii) brain glucose metabolism; (iii) mitochondrial bioenergetic function and dynamics (fusion-fission, biogenesis, degradation and transport); and (iv) synaptic integrity and neurotransmitters were evaluated. Behavioral and cognitive performance was evaluated two weeks before the sacrifice of mice.

## 2. Results

### 2.1. IHC Partially Prevents Behavioral and Cognitive Alterations in 3×Tg-AD Mice

**Figure 1:**
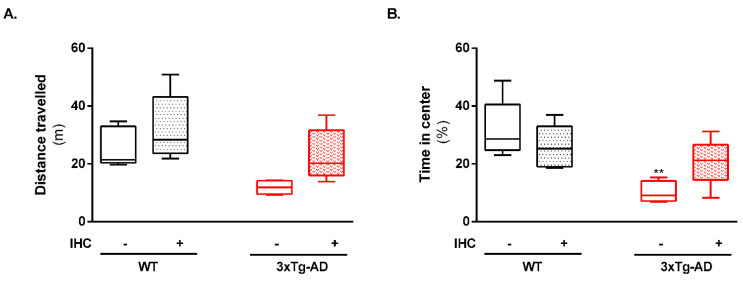
Effect of IHC on locomotor activity and anxiety-like behavior in the 3×Tg-AD mouse model during the open-field test. (**A**) Total distance travelled and (**B**) time spent in the center of the open-field arena during the open field test. Data are presented as box-and-whisker plots representing median and interquartile range (IQR), with minimum and maximum values of 5–9 animals from each experimental condition. Statistical significance: ** *p* < 0.01 when compared with control WT mice.

Ten weeks after the implementation of IHC protocol, the effect of this non-pharmacological strategy on AD-related behavioral and cognitive manifestations was assessed by performing the open-field and Morris water maze (MWM) tests. In the open-field test, the 3×Tg-AD mice exhibited an increased anxiety-like behavior when compared with the control wild-type (WT) mice, as reflected by the decrease in the total distance travelled (Figure 1A) and time spent in the center of the open-field arena (Figure 1B). Notably, IHC was able to avert this anxiety pattern in 3×Tg-AD mice (Figure 1A,B).

**Figure 2:**
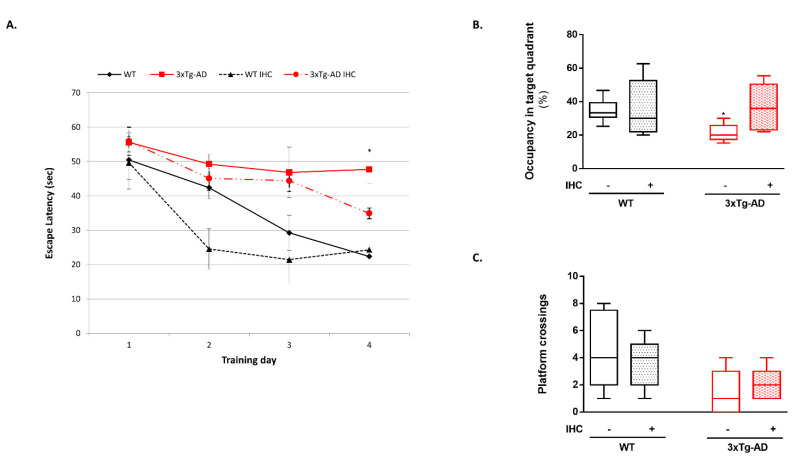
Effect of IHC on learning and spatial memory in the 3×Tg-AD mouse model during the MWM test. (**A**) Mean escape latencies to reach the hidden platform during the training trials, which were conducted for 4 consecutive days. (**B**) The percent time in the target quadrant during the probe trial performed 24 h after the last training trial. (**C**) The number of platform crossings during the probe trial. Data are presented as box-and-whisker plots representing median and IQR, with minimum and maximum values of 5–9 animals from each experimental condition. Statistical significance: * *p* < 0.05 when compared with control WT mice.

As expected, during the acquisition phase of the MWM test, 3×Tg-AD mice presented higher escape latencies to find the hidden platform across the four-day training period when compared with the respective control WT mice (Figure 2A). Furthermore, 3×Tg-AD mice also spent less time in the target quadrant during the retention phase and exhibited a decrease in the number of platform crossings (Figure 2B,C), corroborating the existence of learning and memory deficits in this mouse model of AD. Remarkably, IHC was able to decrease the escape latency time on day 4 of the acquisition phase and increase the time spent in the target quadrant in the retention trial, reflecting a protective effect of IHC against 3×Tg-AD-related cognitive deterioration (Figure 2A,B).

### 2.2. HP Abrogates Aβ Pathology in the Brain Cortex of 3×Tg-AD Mice

**Figure 3:**
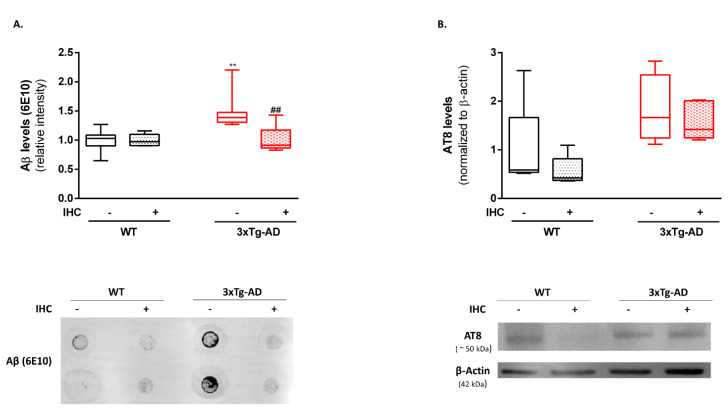
Effect of IHC on AD hallmarks in the brain cortex of 3×Tg-AD mouse model. (**A**) Using the antibody 6E10, the Aβ levels were detected by immuno-dot blot. (**B**) Representative Western blot image and densiometric analysis of tau levels detect with AT8 antibody which recognizes Ser202/Thr205 residues. β-actin was used as an internal loading control. Data are presented as box-and-whisker plots representing median and IQR, with minimum and maximum values of 5 animals from each experimental condition. Statistical significance: ** *p* < 0.01 when compared with control WT mice; ^##^ *p* < 0.01 when compared with 3×Tg-AD mice.

In a step further, the impact of IHC on the neurotoxic proteins of AD-Aβ and phosphorylated tau was evaluated on brain cortical homogenates by immuno-dot-blot and immunoblotting, respectively. Using the 6E10 antibody, which recognizes Aβ monomers, fibrils and oligomers, a significant increase in Aβ levels was detected in the brain cortex of 3×Tg-AD mice when compared with control WT mice. Of note, IHC was able to reduce brain cortical Aβ levels near to those observed in control WT mice (Figure 3A). No significant alterations were observed in the brain cortical levels of AT8, which detects phosphorylated tau protein at Ser202/Thr205 residues, among the experimental groups (Figure 3B).

### 2.3. IHC Ameliorates Mitochondrial Bioenergetic Function in the Brain Cortex of 3×Tg-AD Mice

**Figure 4:**
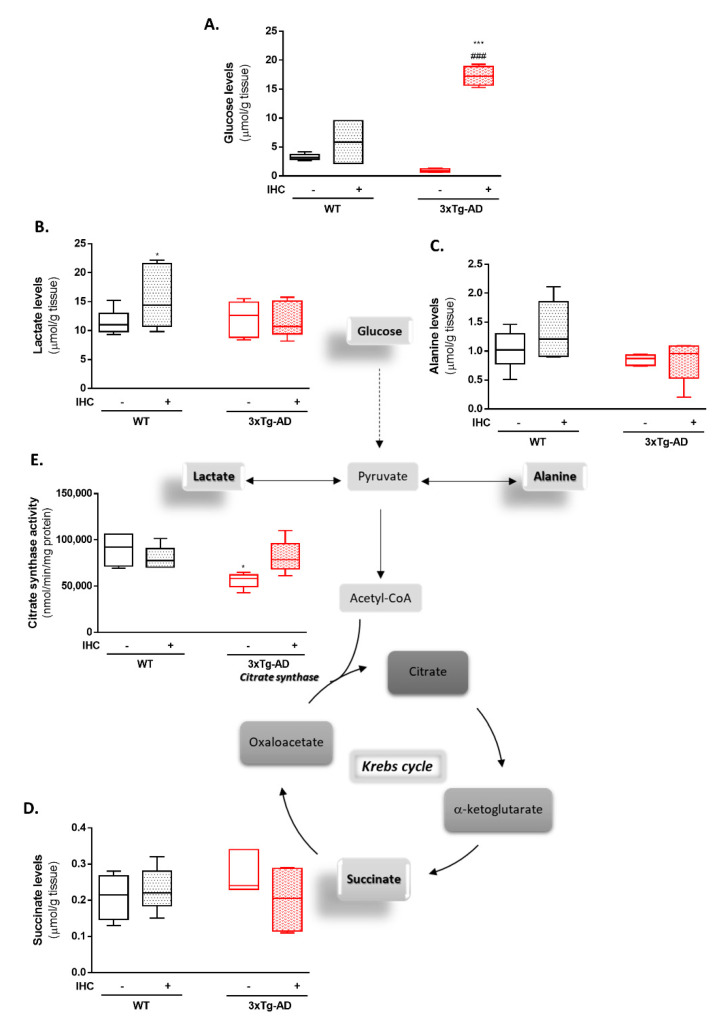
Effect of IHC on energy metabolism in the brain cortex of 3×Tg-AD mouse model. The levels of the metabolites (**A**) glucose, (**B**) lactate, (**C**) alanine, and (**D**) succinate were detected in the brain cortex by NMR spectroscopy. (**E**) Citrate synthase activity was measured spectrophotometrically at 412 nm. Data are presented as box-and-whisker plots representing median and IQR, with minimum and maximum values of 5–7 animals from each experimental condition. Statistical significance: * *p* < 0.05, *** *p* < 0.001 when compared with control WT mice; ^###^ *p* < 0.001 when compared with 3×Tg-AD mice.

Dysfunction of brain glucose metabolism and mitochondrial bioenergetics are early events during the pathological course of AD. Importantly, it has been postulated that energetic crisis is an important driving force underlying neuronal loss and cognitive decline in AD [21,22,23]. Within this scenario, we advocate that IHC might prevent AD-related brain hypometabolism in the 3×Tg-AD mouse model. Therefore, in a first approach the levels of some metabolites involved in glycolysis and Krebs cycle were detected in the brain cortex by ^1^H nuclear magnetic resonance (NMR) spectroscopy and the activity of citrate synthase by spectrophotometry. Interestingly, IHC significantly averted the slight decrease in glucose levels (Figure 4A) and the increase in succinate levels (Figure 4D) in the brain cortex of 3×Tg-AD mice. Furthermore,3×Tg-AD rats also abrogated the significant decrease in the citrate synthase activity in the 3×Tg-AD mice, which is known to be the enzyme that catalyzes the first reaction of Krebs cycle (Figure 4E).

In a step further, using freshly isolated brain cortical mitochondria energized with succinate (substrate for the mitochondrial respiratory Complex II), oxidative phosphorylation capacity and mitochondrial respiration were evaluated by polarographic methods. Regarding oxidative phosphorylation system, IHC was shown to promote a significant increase in ΔΨm and repolarization levels in brain cortical mitochondria from 3×Tg-AD mice (Table 1). As shown in Table 1, IHC further induced a significant increase in mitochondrial respiratory state 2, RCR and FCCP-stimulated respiration. RCR is a measure of the coupling between substrate oxidation and phosphorylation and is a good indicator of mitochondrial integrity. Overall, these data suggest that IHC strongly improves mitochondrial coupling and respiratory efficiency in the brain cortex of 3×Tg-AD mice.

### 2.4. IHC Reduces the Levels of Mitochondrial Fission-Related Proteins in the Brain Cortex of 3×Tg-AD Mice

**Figure 5:**
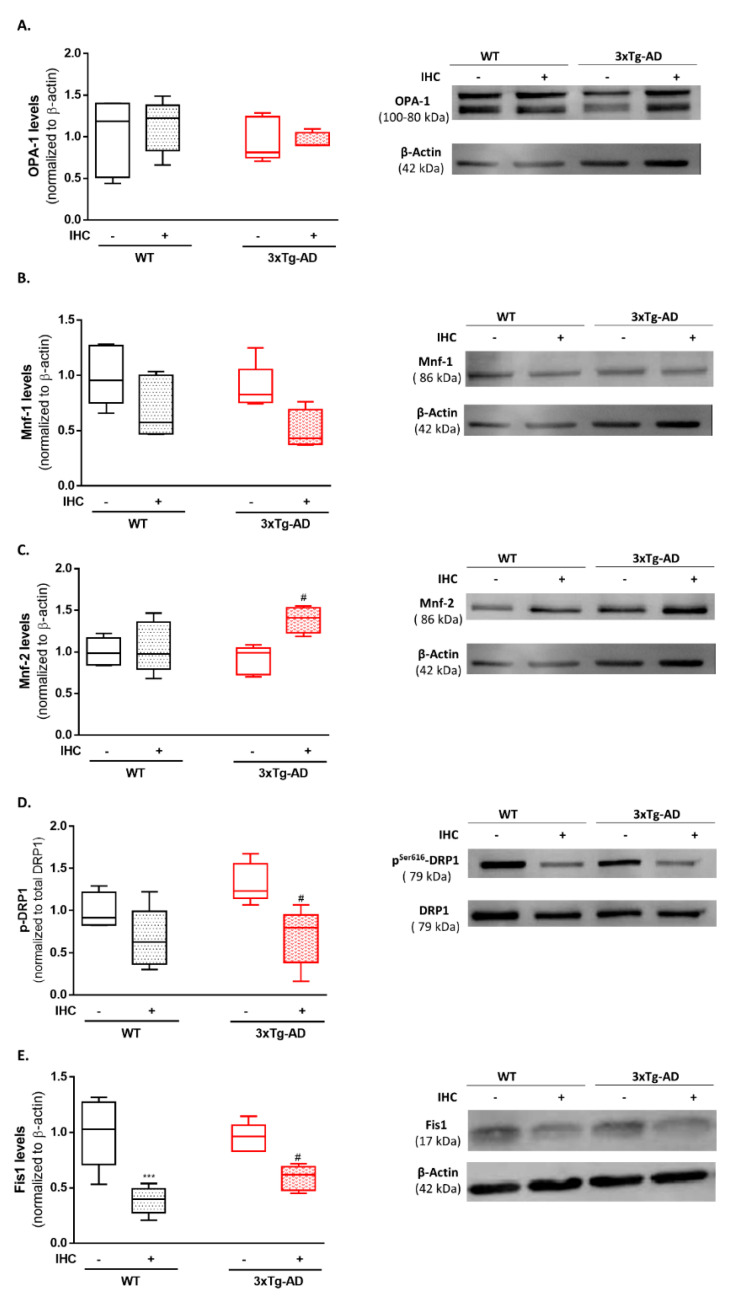
Effect of IHC on mitochondrial fusion-fission machinery in the brain cortex of 3×Tg-AD mouse model. (**A**) Representative Western blot image and densiometric analysis of OPA-1, (**B**) Mnf-1, (**C**) Mnf-2, (**D**) DRP1 (**E**) Fis1 protein levels. β-actin was used as an internal loading control. Active form of DRP1 (p^Ser616^-DRP1) was normalized to total DRP1 levels. Data are presented as box-and-whisker plots representing median and IQR, with minimum and maximum values of 5 animals from each experimental condition. Statistical significance: *** *p* < 0.001 when compared with control WT mice; ^#^ *p* < 0.05 when compared with 3×Tg-AD mice.

In face of this robust improvement of the brain bioenergetic profile, we asked whether the mitochondrial quality control mechanisms could be acting on the protective effect afforded by IHC paradigm against AD phenotype. The mitochondrial network can be reconfigured due to alterations in the metabolic state. It is well-known that mitochondrial network morphology is governed by two opposing processes, mitochondrial fusion and mitochondrial fission. Frequent cycles of fusion and fission contribute to the maintenance of mitochondrial function and optimize bioenergetic capacity [24,25]. Therefore, the next step of this study was to determine by immunoblotting the brain cortical levels of the mitochondrial shaping proteins, optic atrophy protein-1 (OPA-1), mitofusin-1 (Mnf-1) and mitofusin-2 (Mnf-2), which control the mitochondrial fusion process, and fission protein 1 (Fis1) and dynamin-like protein 1 (DRP1), which regulate mitochondrial fission. At nine months of age and compared with the control WT mice, 3×Tg-AD mice only presented a slight increase in the active form of the fission protein DRP1 (p^Ser616^-DRP1) (Figure 5D). Remarkably, IHC induced a significant increase in the levels of mitochondrial fusion protein Mnf-2 (Figure 5C) and a significant decrease in the active form of DRP1 (Figure 5D) and Fis1 (Figure 5E) in the brain of 3×Tg-AD mice, suggesting a shift of mitochondrial network dynamics towards mitochondrial fusion.

### 2.5. IHC Promotes Mitochondrial Biogenesis Increasing Mitochondrial Content in the Brain Cortex of 3×Tg-AD Mice

**Figure 6:**
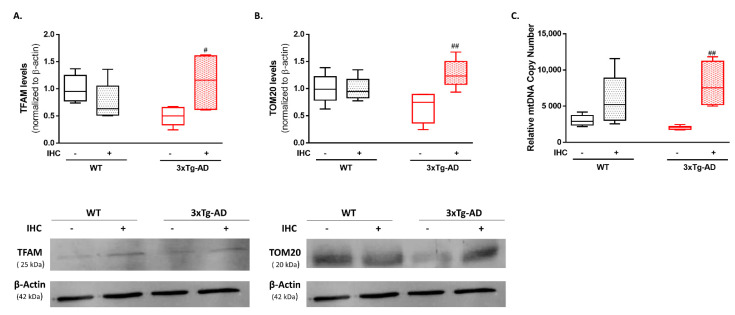
Effect of IHC on mitochondrial biogenesis in the brain cortex of 3×Tg-AD mouse model. (**A**) Representative Western blot image and densiometric analysis of TFAM and (**B**) TOM20 protein levels. β-actin was used as an internal loading control. (**C**) The relative mtDNA copy number was determined by RT-PCR. Data are presented as box-and-whisker plots representing median and IQR, with minimum and maximum values of 5 animals from each experimental condition. Statistical significance: ^#^ *p* < 0.05, ^##^ *p* < 0.01 when compared with 3×Tg-AD mice.

In response to the bioenergetic demand, new mitochondria are de novo synthesized in a process termed mitochondrial biogenesis. To gain further insights on the mitoprotective events underlying the rescuing of mitochondrial bioenergetic profile mediated by IHC, mitochondrial biogenesis was evaluated by assessing the protein levels of TFAM, one of the main effectors that regulate mitochondrial DNA (mtDNA) transcription, and TOM20, a mitochondrial outer membrane protein that functions as a reliable marker of mitochondrial mass. Our results revealed a modest decrease on TFAM and TOM20 protein levels in the brain cortex of 3×Tg-AD mice, when compared with the respective control WT mice (Figure 6A,B). However, IHC was able to significantly increase TFAM and TOM20 protein levels in the brain cortex of 3×Tg-AD mice (Figure 6A,B). To corroborate these findings, mitochondrial mass was also evaluated by determining the mtDNA content by real-time qPCR (RT-PCR). Consistently, IHC was able to induce a significant increment in the relative mtDNA copy number in the brain cortex of 3×Tg-AD mice (Figure 6C).

### 2.6. IHC Enhances the Molecular Machinery Behind Mitophagy in the Brain Cortex of 3×Tg-AD Mice

**Figure 7:**
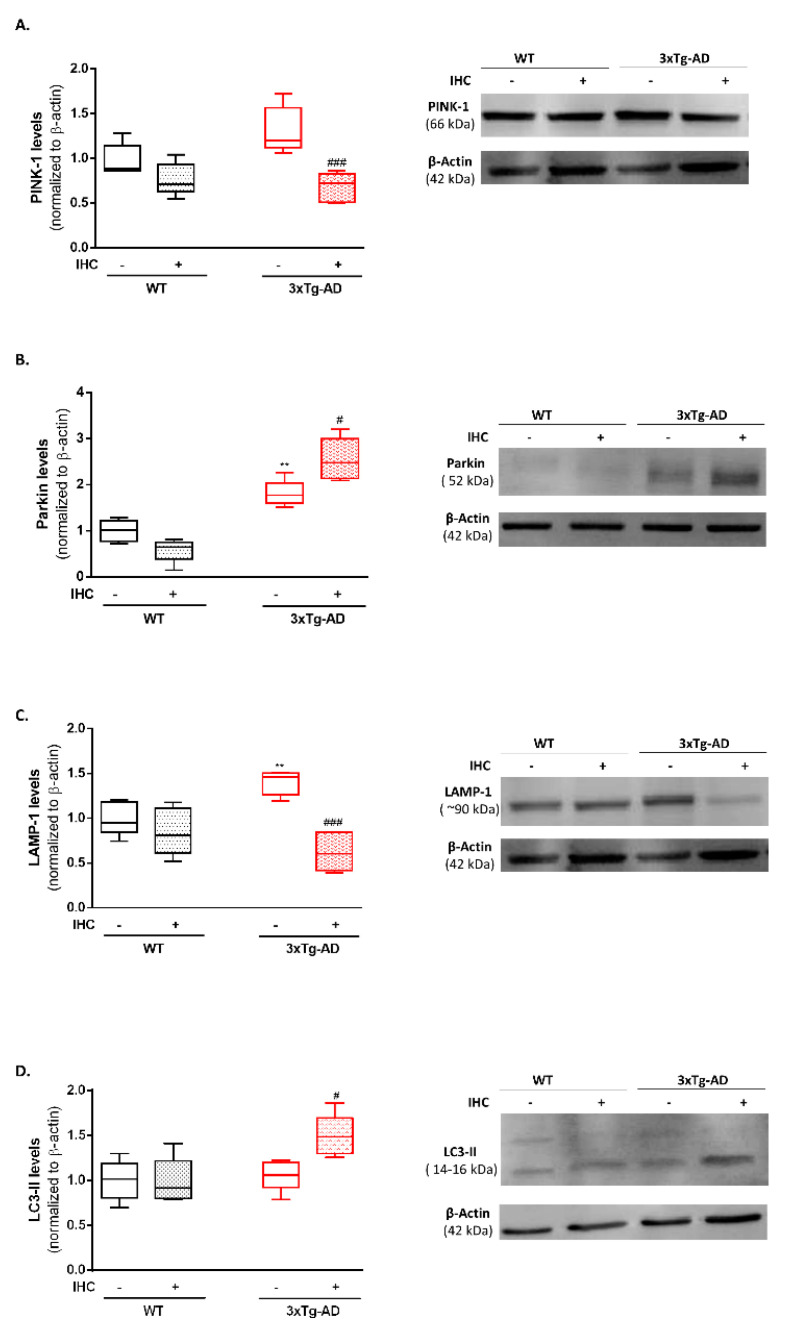
Effect of IHC on mitophagy-related markers in the brain cortex of 3×Tg-AD mouse model. (**A**) Representative Western blot image and densiometric analysis of PINK-1, (**B**) Parkin, (**C**) LAMP-1 and (**D**) LC3-II protein levels. β-actin was used as an internal loading control. Data are presented as box-and-whisker plots representing median and IQR, with minimum and maximum values of 5 animals from each experimental condition. Statistical significance: ** *p* < 0.01 when compared with control WT mice; ^#^ *p* < 0.05, ^###^ *p* < 0.001 when compared with 3×Tg-AD mice.

Besides mitochondrial fusion-fission and mitochondrial biogenesis, the selective degradation of damaged or unwanted mitochondria by mitophagy constitutes another crucial checkpoint of the mitochondrial quality control axis. Briefly, the reduction of ΔΨm function as an “eat me” signal that is sensed by PTEN-induced kinase (PINK1), which in turn recruits Parkin to the damaged mitochondria. Subsequently, active Parkin leads to the ubiquitination of mitochondrial proteins which serves as a distinct molecular code required for the selective recruitment of autophagy machinery and the completion of mitophagy [26,27]. We hypothesized that increased mitophagy could represent an adaptative event underlying the improvement of mitochondrial bioenergetic profile in the 3×Tg-AD mice submitted to the IHC protocol. To this end, the protein levels of PINK-1, Parkin, Lysosomal-associated membrane protein 1 (LAMP-1, a standard marker for lysosomes) and microtubule-associated protein light chain 3-II (LC3-II, a standard marker for autophagosomes) were detected by immunoblotting. In comparison with the control WT mice, 3×Tg-AD mice presented a significant increase in Parkin and LAMP-1 protein levels (Figure 7B,C), suggesting a failure during the mitophagic process. However, our results revealed that IHC promoted a significant increase in Parkin and LC3-II protein levels (Figure 7B,D) and a significant decrease in PINK-1 and LAMP-1 protein levels (Figure 7A,C), fostering the idea that IHC promotes the efficient removal of dysfunctional mitochondria in order to sustain mitochondrial bioenergetics.

### 2.7. IHC Increases Mitochondrial Motor Proteins in the Brain Cortex of 3×Tg-AD Mice

**Figure 8:**
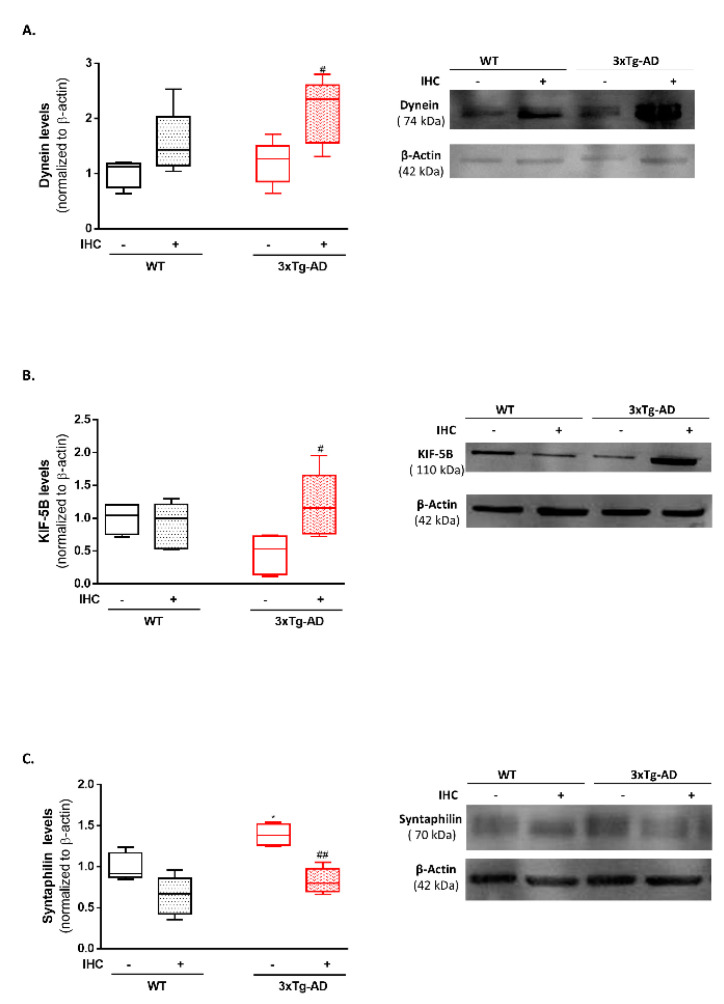
Effect of IHC on mitochondrial transport-related proteins in the brain cortex of 3×Tg-AD mouse model. (**A**) Representative Western blot image and densiometric analysis of dynein, (**B**) KIF-5B and (**C**) syntaphilin protein levels. β-actin was used as an internal loading control. Data are presented as box-and-whisker plots representing median and IQR, with minimum and maximum values of 5 animals from each experimental condition. Statistical significance: * *p* < 0.05 when compared with control WT mice; ^#^ *p* < 0.05, ^##^ *p* < 0.01 when compared with 3×Tg-AD mice.

The vigorous transport of mitochondria in both anterograde and retrograde directions is crucial to distribute functional mitochondria according to the energetics requirements of the different neuronal compartments and to remove dysfunctional mitochondria, respectively [28,29]. In this sense, to gain further insights into the long-lasting mitoprotective effects of IHC phenomenon, the levels of the motor proteins dynein and kinesin family member 5B (KIF-5B) and the mitochondrial-anchoring protein syntaphilin were detected in the brain cortex. The 3×Tg-AD mice exhibited a slight decrease in KIF-5B protein levels (Figure 8B) and a significant increase in syntaphilin protein levels (Figure 8C), when compared with the respective control WT mice. Notably, IHC was able to significantly increase both dynein and KIF-5B and decrease syntaphilin protein levels in the brain cortex of 3×Tg-AD mice (Figure 8A–C) suggesting that IHC enhances the bidirectional transport of mitochondria, favoring the distribution of new mitochondria to the synaptic terminal and mitophagy.

### 2.8. IHC Downregulates SNAP-25 and Increases Glutamate and GABA Levels and in the Brain Cortex of 3×Tg-AD Mice

**Figure 9:**
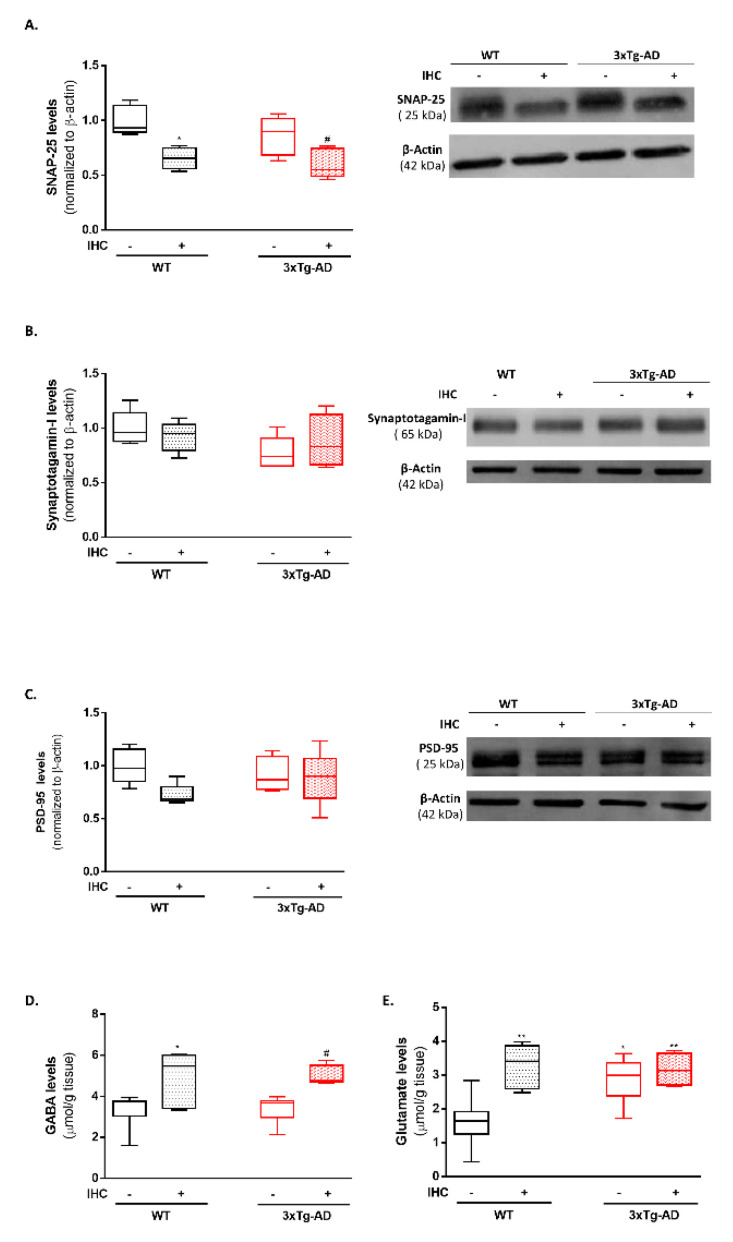
Effect of IHC on synaptic integrity markers and neurotransmitters levels in the brain cortex of 3×Tg-AD mouse model. (**A**) Representative Western blot image and densiometric analysis of SNAP-25, (**B**) synaptotagamin-I and (**C**) PSD-95 protein levels. β-actin was used as an internal loading control. The levels of the neurotransmitters (**D**) GABA and (**E**) glutamate were detected in the hippocampus by NMR spectroscopy. Data are presented as box-and-whisker plots representing median and IQR, with minimum and maximum values of 5 animals from each experimental condition. Statistical significance: * *p* < 0.05, ** *p* < 0.01 when compared with control WT mice; ^#^ *p* < 0.05, ^##^ *p* < 0.01 when compared with 3×Tg-AD mice.

Given the importance of mitochondrial network dynamics for the maintenance of synaptic integrity and functioning, in this last set of experiments the levels of the pre-synaptic proteins, SNAP-25 and synaptotagmin-I, and the post-synaptic density protein-95 (PSD-95) were evaluated by immunoblotting. As shown in Figure 9B, only a modest decrease in synaptotagmin-I was detected in the brain cortex of the 3×Tg-AD mice, when compared with the respective control WT mice. Surprisingly, IHC promoted a significant decrease in SNAP-25 protein levels in the brain cortex of WT and 3×Tg-AD mice (Figure 9A). This result raises the possibility that the reduction in SNAP-25 may constitute an adaptive response triggered by IHC to sustain synaptic and neuronal integrity and, thus preserve cognitive functions.

Lastly, the brain cortical levels of the neurotransmitters GABA and glutamate were also measured by NMR spectroscopy. When compared with the control WT mice, 3×Tg-AD mice exhibited a significant increase in glutamate levels (Figure 9E). Interestingly, IHC induced a significant increase in the levels of GABA and glutamate on both WT and 3×Tg-AD mice (Figure 9D,E).

## 3. Discussion

**Figure 10:**
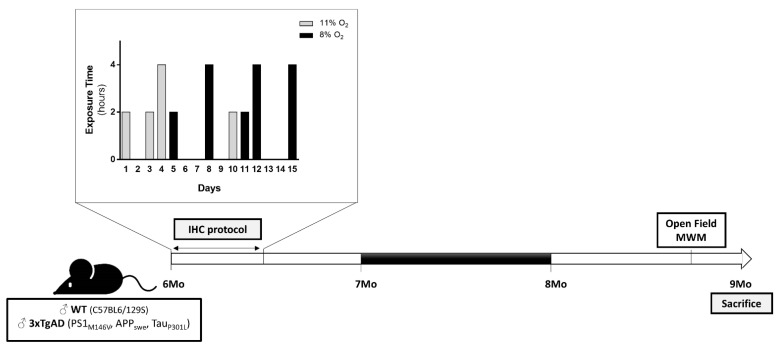
Experimental design. 6-month-old male WT and 3×Tg-AD mice were randomly divided into two groups: (i) a conditioned and (ii) a non-conditioned control group. In the IHC paradigm, mice were exposed to 9 hypoxic episodes over 2 weeks for either 2 or 4 h at either 8% or 11% O_2_ during a 2-week period. Open-field and MWM tests were performed around 8.5-month-old. Mice were euthanized at 9 months of age.

Dovetailing in the old saying “what doesn’t kill you makes you stronger”, conditioning is known to trigger an adaptive reprogramming of the brain, enhancing the resilience to disease conditions. Within this scenario, the present study was designed to extend the knowledge regarding the potential protective impact of IHC against AD-related phenotype, putting a special focus on the different aspects of mitochondrial (patho)biology. Taking advantage from the 3×Tg-AD mouse model of AD, this study revealed that the implementation of IHC protocol during a two-week period (Figure 10) was able to confer long-term protective effects on behavior and cognitive performance, Aβ pathology and mitochondrial bioenergetic function. Interestingly, the boost on mitochondrial bioenergetic profile was accompanied by: i) a reduction in mitochondrial fission proteins DRP1 and Fis1 and an increase in mitochondrial fusion protein Mnf-2; ii) stimulation of mitochondrial biogenesis and mitophagy; and iii) modulation of the bidirectional transport of mitochondria as denoted by the increase in motor proteins dynein and KIF-5B.

One of the major challenges for the scientific community is to develop a “magic pill” able to halt and/or reverse AD-related behavioral disturbances and cognitive decline. As aforementioned, the conundrum for AD therapy concerns with the fact that the degenerative events behind the selective neuronal loss precede the clinical signs of the disease by decades [6]. In this study, a IHC protocol was employed to 6-month-old 3×Tg-AD mice, which already exhibit AD-like symptomatology at this age [30]. Remarkably, this non-pharmacological procedure was able to avert the anxiety behavior and the memory and learning deficits in 3×Tg-AD mice (Figure 1 and Figure 2), as mirrored by longer time spent in the center of the open-field arena and lower escape latencies to reach the hidden platform and by the increased time spent in the target quadrant in the retention trial of the MWM test. Similarly, using a non-transgenic rat model of AD, Manukhina and collaborators [16] found that IHC (14 days, 4 h per day, simulating an altitude of 4000 m) protected cognitive functions by blocking memory degradation assessed 14 days after Aβ administration. In the same line, IHC was also shown to improve cognitive performance and reduce anxiety-related behavior in APP/PS1 mice [31]. Besides these promising results in rodent models of AD, recent breakthroughs also revealed that intermittent hypoxia training improves short-term memory and attention in elderly patients with amnestic mild cognitive impairment (aMCI), often considered a prodromal phase of AD [32].

Despite the mechanisms underlying AD onset remain puzzling, scientific efforts suggest that Aβ and phosphorylated tau are important triggers, mediators and aggravators of AD pathology, acting in both separate and synergistic ways to the degenerative events that occur in AD brain [33,34,35]. Notably, our results revealed that IHC was able to significantly reverse Aβ levels in the brain cortex of nine-month-old 3×Tg-AD mice (Figure 3A). In the same line, Meng and collaborators [31] observed that IH exposure significantly reduced the number of Aβ plaques in the brain cortex and hippocampus of APP/PS1 mouse model. On the flipside, a study performed in 2013 demonstrated that chronic intermittent hypoxia/reoxygenation (5% O_2_ and 21% O_2_ every 10 min, 8 h/day for four weeks) favors the generation and accumulation of Aβ42 in the brain of six-month-old 3×Tg-AD mice [36]. Concerning tau pathology, no alterations in AT8 levels were observed among the experimental groups (Figure 3B). However, compelling evidence points out that intermittent hypoxia differentially modulates endogenous tau phosphorylation [37,38]. Overall, these data draw attention to the fact that intermittent hypoxia can have distinct effects depending on the nature of the hypoxic episodes (i.e., severity, duration, number of episodes per day).

The early stages of AD are marked by a hypometabolic state, as noticed by hampered brain glucose metabolism [39], impaired activities of the Krebs cycle enzymes [40,41], and defects in the mitochondrial electron transport chain [42,43,44,45] with the consequent depletion of ATP levels. Lessons from clinical and experimental studies advocate that disturbances in brain energy metabolism are due to a convergence of Aβ and tau on mitochondria, favoring a vicious cycle of deleterious events [46]. Back to 2012, data from our laboratory demonstrated that brain mitochondria from 11-month-old 3×Tg-AD mice are characterized by and impaired bioenergetic function, decreased Ca^2+^ buffering capacity and ultrastructural abnormalities [47]. In this study, IHC phenomenon was shown to rescue brain energy metabolism in 3×Tg-AD mice as denoted by the increase in brain cortical levels of glucose and citrate synthase activity (Figure 4). Furthermore, IHC also abolished the defects in oxidative phosphorylation system and mitochondrial respiratory function detected in brain cortical mitochondria isolated from 3×Tg-AD mice (Table 1). Of note, IHC was able to restore ΔΨm and RCR and to significantly increase FCCP-stimulated respiration, suggesting that IHC strongly boost mitochondrial bioenergetic capacity. Consistently, IHC was shown to protect brain mitochondrial function, namely cytochrome *c* oxidase activity in other models of brain disorders [48,49]. However, whether IHC improves mitochondrial bioenergetic function remains unclear. Are mitochondrial quality and quantity control mechanisms targeted by this non-pharmacological strategy?

Aside from the well-characterized metabolic fingerprint, it has been documented a massive accumulation of mitochondria with an altered morphology in dystrophic neurites during the pathological course of AD [28,50,51]. Considering the dynamic nature of mitochondria, it has been proposed that this overcrowding of mitochondria with a defective morphology result from the catastrophic combination of excessive mitochondrial fission, impaired mitochondrial biogenesis, inefficient mitophagy, and faulty mitochondrial transport [52]. Indeed, fluctuations in brain metabolic status are often accompanied by a remodeling of the mitochondrial network [53]. Mitochondrial morphology is controlled by the fine-tunning balance between opposing fusion and fission events. A shift towards mitochondrial fusion favors the generation of an interconnected mitochondrial network, whereas a shift towards mitochondrial fission produces multiple mitochondrial fragments [54]. Using postmortem hippocampal tissue from AD individuals, a pioneer study from Wang and collaborators [55] reported reduced levels of the mitochondrial fusion proteins OPA-1, Mnf-1 and Mnf-2. In AD cybrids it was observed increased translocation of the mitochondrial fission DRP1 to mitochondria and reduced Mnf-2 protein levels, culminating in the fragmentation of the mitochondrial network [56]. Consistently, a recent study reported an increase in the active form of DRP1 (p^Ser616^-DRP1) in the brain cortex and hippocampus of 10-month-old female 3×Tg-AD mice, bolstering the idea that AD is marked by excessive mitochondrial fission [57]. Our results revealed only a modest increase in the active form of DRP1 in the brain cortex of 3×Tg-AD mice (Figure 5D). However, IHC induced a significant increase in Mnf-2 levels and a notorious decrease in both DRP1 and Fis1, suggesting that IHC favors mitochondrial fusion. Under conditions of moderate cellular stress, increased fusion, forming a hyperfused meshwork constitute an adaptive strategy to preserve metabolic homeostasis and cell survival [58]. Notably, mitochondrial fusion is an important mechanism that allows the exchange of lipid membrane and intramitochondrial contents between different mitochondria to enable mtDNA repair and equally distribute metabolites to maintain a healthy mitochondrial population [59]. In this sense, it is alluring to speculate that the shift in mitochondrial fusion-fission machinery towards fusion induced by IHC represents an adaptive mechanism to restore mitochondrial bioenergetic function in 3×Tg-AD mice.

Mitochondrial bioenergetic function is also reliant on mitochondrial mass and turnover. Therefore, another potential explanation for the long-lasting protective effect of IHC on mitochondrial bioenergetics in 3×Tg-AD mice may also rely on the stimulation of mitochondrial biogenesis. Compromised mitochondrial biogenesis, manifested by reduced peroxisome proliferator activator receptor gamma-coactivator 1α (PGC-1α) and nuclear respiratory factors (NRFs) levels and mtDNA content has been documented in human AD brain tissue and in in vitro and in vivo models of the disease [60,61]. Consistently, our results showed a modest reduction in TFAM and TOM20 levels and mtDNA content in the brain cortex of 3×Tg-AD mice (Figure 6). Remarkably, these alterations were abrogated by IHC phenomenon. In line with this finding, transient hypoxia was previously demonstrated to activate a nuclear-encoded regulatory program that stimulates mitochondrial biogenesis [62]. Consequently, this program led to an increase in mtDNA transcription and content, followed by structural evidence of neuronal mitochondrial biogenesis (increased neuronal mitochondria number and/or volume density) [62].

Side by side with mitochondrial biogenesis, the selective removal of damaged or unwanted mitochondria by mitophagy also represents an important “mitocheckpoint” responsible for the maintenance of mitochondrial quantity and quality [63]. Evidence from the literature revealed that AD progression is associated with the aberrant accumulation of undesirable and irreparable damaged mitochondria due to a failure in the recruitment of the autophagic components to mitochondria [64]. Mitophagy failure in sporadic AD is related with a defective labeling of mitochondria to be degraded by mitophagy, whereas in familial AD, mitochondria are correctly tagged for recycling but a deficiency in the degradation step of the mitophagic process occurs [65,66]. In our mice model of familial AD, a significant increase in Parkin (Figure 7B) and LAMP-1 levels (Figure 7C) was detected, suggesting an increase in lysosomal mass due to a failure in the degradation phase of mitophagy. IHC induced a significant increase in Parkin (Figure 7B) and LC3-II levels (Figure 7D) and a reduction in PINK-1 (Figure 7A) and LAMP-1 levels (Figure 7C), indicating that IHC facilitates the efficient removal of dysfunctional mitochondria. Similarly, a previous study demonstrated that conditioning paradigm is able to mitigate ischemia-triggered brain damage by promoting Parkin-dependent mitophagy [67].

Mitochondrial trafficking is another dynamic property of mitochondrial biology, being essential to distribute new and functional mitochondria according to the energetic requirements (anterograde transport) and to transport irreversible damaged mitochondria in the retrograde direction towards the soma to be degraded by mitophagy [28]. Faulty anterograde and retrograde transport has been shown to contribute to mitochondrial overcrowding, mislocalization and bioenergetic failure in AD [68,69]. Nine-month-old 3×Tg-AD mice only presented a slight decrease in the brain cortical levels of the motor protein KIF-5B (Figure 8B). However, IHC was able to induce a significant increase in both dynein (Figure 8A) and KIF-5B levels (Figure 8B) in 3×Tg-AD mice. Indeed, hypoxia has been identified as one of the few bona fide stimuli to induce a shift in the distribution of the mitochondrial network [70]. Furthermore, IHC also prevented the significant increase in syntaphilin levels (Figure 8C) in 3×Tg-AD mice. Syntaphilin is a “static anchor” that immobilizes mitochondria, leading to the arrest of mitochondria at specific locations with high energy demands [71,72,73]. More recently, it was demonstrated that the release of syntaphilin removes stressed mitochondria from axons in the context of AD pathology, which contributes to the refurbishment of axonal mitochondrial quality [74]. In the face of these findings, we advocate that IHC facilitates the bidirectional transport of mitochondria to support the increase in mitochondrial biogenesis and mitophagy, culminating in enhanced mitochondrial bioenergetics in 3×Tg-AD mice.

Synaptic failure is assumed to be an early progressive pathological event in AD, resulting in impaired cognitive function and memory loss, which is particularly noticeable at later stages of disease [75]. Recent breakthroughs outlined a close association between altered mitochondrial dynamics and the occurrence of synaptic lesions and degeneration in AD neurons [76]. In some way this is not surprising considering that synaptic terminals are high energy-demanding structures that rely on mitochondria-derived ATP to maintain functional neurotransmission [72]. Particularly, a region-specific depletion of synaptic mitochondria was observed in the brain of AD individuals, reinforcing the idea that defective mitochondrial transport and dynamics contributes to “synaptic starvation” and neuronal loss in AD [77]. In accordance, disruption of glutamatergic and GABAergic neurotransmission and neuronal circuits has also been extensively documented in the AD brain [78,79,80]. Hitherto, it was documented premature alterations in synaptic plasticity in presymptomatic three-month-old 3×Tg-AD mice [81]. Additionally, it was also reported that 11-month-old 3×Tg-AD mice exhibit alterations in synaptic integrity markers, particularly a marked decreased in PSD-95 levels in the brain cortex and a reduction in SNAP-25 levels in the hippocampus [82]. In this study, only a slight decrease in the levels of the pre-synaptic marker synaptotagamin-I (Figure 9B) and a significant increase in glutamate levels (Figure 9B) were detected in the brain cortex of 3×Tg-AD. Unexpectedly, our results revealed that IHC induced a marked reduction in SNAP-25 levels (Figure 9A) and a significant increase in both GABA and glutamate levels (Figure 9D,E) in the brain cortex of WT and 3×Tg-AD mice. In face of this data, an exciting question has emerged: could IHC trigger a long-lasting remodeling of the synaptic structure and microenvironment to sustain neuronal and cognitive function? As a matter of fact, it was previously demonstrated that hypoxic conditioning significantly affects synaptic curvature and the percentage of synapses with presynaptic mitochondria [83]. Furthermore, IHC was also shown to be able to rescue spatial learning and memory deficits by inducing functional synaptogenesis via brain-derived neurotrophic factor (BDNF) expression [84]. Additionally, an outstanding study found that hypoxia downregulates SNAP-25, resulting in reduced vesicular docking and synaptic remodeling in order to circumvent excitotoxic neurodegeneration [85].

We demonstrated herein that IHC effectively counteracts the behavioral and cognitive abnormalities, Aβ levels increase and mitochondrial bioenergetic deficits in the 3×Tg-AD mouse model in part by targeting mitochondrial quality control axis. However, an open question from this study is how long does the beneficial impact of this non-pharmacological and non-invasive procedure last? Furthermore, a potential gap of this study pertains with the fact that sex/gender dimension was not addressed taking into account the disparities both in AD prevalence and severity between man vs woman. Therefore, further studies are warranted to advance our in-depth understanding on the (mito)protective events behind IHC phenomenon and if they hold a translational promise and whether IHC can be employed as a preventive/adjuvant therapy to counteract AD symptomatology and neuropathology in a timely manner.

## 4. Materials and Methods

### 4.1. Animals and Ethics Statements

3×Tg-AD mice harboring PS1/M146V, APPswe, and tauP301L transgenes, and respective WT control mice with the same genetic background (C57BL6/129S) were bred and maintained at Center for Neuroscience and Cell Biology (CNC) Animal House Facility (license n° 520.000.000.2006, from the Portuguese Animal Welfare authorities) and provided ad libitum access to food and water and maintained under controlled light (12 h day/night cycle), temperature (22–24 °C) and humidity (50–60%). The 6-month-old male WT and 3×Tg-AD mice were randomly divided into two groups: i) a conditioned and ii) a non-conditioned control group. In the IHC paradigm, mice were submitted to a 2-week protocol previously described by Stowe and collaborators [86]. Briefly, mice were exposed to 9 hypoxic episodes over 2 weeks for either 2 or 4 h at either 8% or 11% O_2_ as illustrated in Figure 10. Non-conditioned WT and 3×Tg-AD mice were handled in the same manner, but exposed only to room air. Behavioral analyses were performed around 8.5 months of age during the dark cycle. Mice were euthanized at 9 months of age by cervical dislocation and decapitation and brains were immediately removed and cortices dissected. All procedures were performed to minimize exposure to stress and suffering, in accordance with the animal welfare guidelines of the institutional animal house facility and European and Portuguese legislation (Directive 2010/63/ EU; DL113/2013, August 7).

### 4.2. Behavioral Analyses

Mice were allowed to acclimate to the behavioral testing room 1 h prior to each test. Behavioral tests were performed in consecutive days, by experienced observers blind to the experimental conditions.

#### 4.2.1. Open Field Test

The open field test is a well-established paradigm for assaying locomotion and anxiety-related behaviors [87]. Mice were placed individually in the center of an open field squared arena (and exploration was recorded for a 10-min period using the Stoelting ANY-MAZE video tracking system (Stoelting Co., Wood Dale, IL, USA). The total distance travelled and time spent in the center of the open-field arena were measured.

#### 4.2.2. MWM Test

The MWM test was employed to assess hippocampal-dependent spatial memory as previously described by Morris [88] with slight modifications. The MWM is a swimming-based model in which mice learn to escape a pool of water (120 cm in diameter, 80 cm in height, filled to a depth of 60 cm with water at 23 ± 2 °C) by a hidden platform. Starting points were marked on the outside of the pool as north (N), south (S), east (E) and west (W). Four distant cues were placed 30 cm above the upper edge of the water tank and the position of each symbol marked the midpoint of the perimeter of a quadrant (circle = NE quadrant, square = SE quadrant, cross = SW quadrant and diamond = NW quadrant). Mice were trained for 4 consecutive days with 4 trials per day. For each trial, mice were placed into the pool at one of the 4 predefined starting points in a pseudorandom order and allowed to search for the platform. Mice were guided to the platform if they were unable to reach it by themselves within 60 s. A minimum interval of 15 min was given to the animals between each trial. 24 h after the completion of the acquisition trials, the mice received a single 60 s probe trial. Mice received this probe trial with the platform removed from the pool for testing the functioning of long-term memory. At this time, the percentage of time spent in the target area and the number of platform crossings were recorded with the ANY-MAZE™ video tracking system (Stoelting Co., Wood Dale, IL, USA).

### 4.3. NMR Spectroscopy

A combined extraction of polar and apolar metabolites was performed in brain cortex [89] and the aqueous phase containing the water-soluble metabolites was lyophilized. NMR spectra were acquired by standard methods used in our laboratory [77]. Briefly, 1H-NMR spectra were acquired at 14.1 T, 25 °C, using a Bruker Avance III 600 MHz spectrometer equipped with a QCI cryoprobe. The spectra were acquired with solvent-suppression and a sweep width of 6 kHz, using a delay of 14 s, a water presaturation of 3 s, a pulse angle of 45°, an acquisition time of 3.5 s and at least 128 scans. Sodium fumarate (final concentration of 1 mM) was used as an internal reference (6.50 ppm) to quantify the following metabolites whenever present in solution (multiplet, ppm): lactate (doublet, 1.33); alanine (doublet, 1.45); glutamate (multiplet, 2.02); GABA (triplet, 2.28); succinate (singlet, 2.39); H1-α-glucose (doublet, 5.22). The relative areas of 1H-NMR resonances were quantified using the curve-fitting routine supplied with the NUTSproTM NMR spectral analysis program (Acorn, NMR Inc., Fremont, CA, USA.).

### 4.4. Preparation of Mitochondrial Fraction

Brain cortical mitochondria were isolated from mice by the method of Moreira and collaborators [90], using 0.02% digitonin to free mitochondria from the synaptosomal fraction. Briefly, after animal decapitation, the hippocampus was immediately separated and homogenized at 4 °C in 10 mL of isolation medium (225 mM mannitol, 75 mM sucrose, 5 mM HEPES, 1 mM EGTA, 1 mg/mL BSA, pH 7.4) containing 5 mg of the bacterial protease (Sigma-Aldrich, St. Louis, MO, USA). Single brain homogenates were brought to 30 mL and then centrifuged at 2500 rpm (Sorvall Evolution RC Superspeed Refrigerated Centrifuge) for 5 min. The pellet, including the fluffy synaptosomal layer, was resuspended in 10 mL of the isolation medium containing 0.02% digitonin and centrifuged at 10,000 rpm for 10 min. The brown mitochondrial pellet without the synaptosomal layer was resuspended again in 10 mL of medium and centrifuged at 10,000 rpm for 5 min. The pellet was resuspended in 10 mL of washing medium (225 mM mannitol, 75 mM sucrose, 5 mM HEPES, pH 7.4) and centrifuged at 10,000 rpm for 5 min. The final mitochondrial pellet was resuspended in the washing medium and the protein amount determined by the Biuret method calibrated with BSA [91].

### 4.5. Measurements of ΔΨm

ΔΨm was monitored by evaluating the transmembrane distribution of the lipophilic cation TPP^+^ (tetraphenylphosphonium) with a TPP^+^-selective electrode as previously described [90,92] using an Ag/AgCl-saturated electrode (Tacussel, model MI 402) as reference. TPP^+^ uptake has been measured from the decreased TPP^+^ concentration in the medium sensed by the electrode. The potential difference between the selective electrode and the reference electrode was measured with an electrometer and recorded continuously in a Linear 1200 recorder. The voltage response of the TPP^+^ electrode to log[TPP^+^] was linear with a slope of 59 ± 1, which is in a good agreement with the Nernst equation. Reactions were carried out in a chamber with magnetic stirring in 1 mL of the standard medium (100 mM sucrose, 100 mM KCl, 2 mM KH_2_PO_4_, 5 mM Hepes and 10 μM EGTA; pH 7.4) containing 3 μM TPP^+^. The ΔΨm was estimated by the equation: ΔΨm (mV)=59 log(*v/V*)−59 log(10ΔE/59−1). v, V, and ΔE stand for mitochondrial volume, volume of the incubation medium and deflection of the electrode potential from the baseline, respectively. This equation was derived assuming that TPP^+^ distribution between the mitochondria and the medium follows the Nernst equation, and that the law of mass conservation is applicable. A matrix volume of 1.1 μL/mg protein was assumed. No correction was made for the “passive” binding contribution of TPP^+^ to the mitochondrial membranes, because the purpose of the experiments was to show relative changes in potentials rather than absolute values. As a consequence, we can anticipate a slight overestimation on ΔΨm values. However, the overestimation is only significant at ΔΨm values below 90 mV, therefore, far from our measurements. Mitochondria (0.8 mg/mL) were energized with 5 mM succinate (substrate of complex II) in the presence of 2 μM rotenone in order to activate the mitochondrial electron transport chain. After a steady-state distribution of TPP^+^ had been reached (ca. 1 min of recording), ΔΨm fluctuations were recorded.

### 4.6. Mitochondrial Respiration Measurements

Oxygen consumption of mitochondria was registered polarographically with a Clark oxygen electrode connected to a suitable recorder in a thermostated water-jacketed closed chamber with magnetic stirring [93]. The reactions were carried out at 30 °C in 1 mL of the standard medium (100 mM sucrose, 100 mM KCl, 2 mM KH_2_PO_4_, 5 mM Hepes and 10 µM EGTA, pH 7.4) with 0.8 mg of protein. States 4 and 3 respiration were initiated with 5 mM succinate in the presence of 2 μM rotenone (mitochondrial complex II energization). Exogenous ADP (155 nmol/mg protein) was added to initiate state 3. In some experiments, oligomycin (2 μg/mL) and FCCP (1 μM) were also added to inhibit passive flux through the ATP synthase and to uncouple respiration, respectively.

### 4.7. Citrate Synthase Activity

Citrate synthase activity was determined according to the method previously described by Coore and collaborators [94]. Briefly, 10 μg of brain cortical homogenates were incubated at 37 °C in a reaction buffer containing 100 mM Tris pH 8.0 plus 200 μM Acetyl-CoA, 200 μM 5,5′-dithiobis-2-nitrobenzoic acid. The reaction was started by the addition of 100 μM freshly-prepared oxaloacetate, and followed at 412 nm at 37 °C (ε = 13.6 mM/cm).

### 4.8. Determination of Mitochondrial DNA Copy Number

RT-PCR analysis was performed to determine the mtDNA copy number of the experimental groups as described by Fuke and collaborators [95] with slight modifications. Relative quantification of mtDNA levels was determined by the ratio of the mitochondrial ND1 (mt-Nd1) gene to the single-copy, nuclear-encoded beta-2-microglobulin (β-2MG) gene. Reactions were carried out in an iQ5 system (Bio-Rad), and efficiency of the reactions was determined for the selected primers using serial dilutions of DNA samples. Primer concentration and annealing temperature were optimized, and the specificity of the amplicons was determined by melting curve analysis. The reactions mixture consisted of Maxima SYBR Green qPCR Master Mix (Fermentas -Thermo Fisher Scientific, Rockford, IL, USA), sense and antisense primers (Table 2), and 20 ng of DNA. Each reaction was run in triplicate to calculate relative mtDNA copy number. Ct values of all samples were within the linear range. Ct value differences were used to quantify mtDNA copy number relative to the beta-2-microglobulin gene with the following equation: Relative copy number = 2 ΔCt, where ΔCt is Ctβ2MG -CtND1.

### 4.9. Western Blot Analysis

Brain cortical samples were homogenized in ice-cold lysis buffer (20 mM Tris-HCl pH 7.5, 150 mM NaCl, 1 mM Na_2_EDTA, 1 mM EGTA, 1% Triton, 2.5 mM sodium pyrophosphate, 1 mM β-glycerophosphate, 1 mM Na_3_VO_4_, 1 µg/mL leupeptin) supplemented with 0.1 M phenylmethanesulfonylfluoride (PMSF), 0.2 M dithiothreitol (DTT) and protease and phosphatase inhibitors cocktails (Roche Applied Science, Indianapolis, IN, USA). The homogenates were frozen and defrozen 3 times to favor disruption, centrifuged at 14,000 rpm (Eppendorf centrifuge 5415C) for 10 min at 4 °C and the supernatant collected and stored at −80 °C. Protein concentration was determined by the bicinchoninic acid (BCA) protein assay using the BCA kit (Pierce Thermo Fisher Scientific, Rockford, IL. USA).

Equivalent amounts of protein (40 µg) were resolved by electrophoresis in 8–15% sodium dodecyl sulfate (SDS)-polyacrylamide gels and transferred to polyvinylidene fluoride (Millipore, Billerica, MA, USA) membranes. Non-specific binding was blocked by incubation with blocking buffer [10% BSA in Tris-buffered saline (TBS)] for 1 h at room temperature, with gentle agitation. The blots were subsequently incubated overnight at 4 °C with gentle agitation with the specific primary antibodies. Blots were washed 3 times (5 min each), with TBS containing 0.1% Tween (TBS-T) and then were incubated with the secondary antibodies for 1 h at room temperature with gentle agitation. After membrane incubation with enhanced chemifluorescence reagent (ECF), images were obtained in a VersaDoc Imaging System (Bio-Rad, Hercules, CA, USA) and the density of protein bands calculated using the Quantity OneProgram (Bio-Rad, Hercules, CA, USA). The antibodies used are listed in Table 3.

### 4.10. Immuno-Dot-Blot Assay

Aβ levels were detected using 6E10 antibody in non-denaturing immuno-dot blot conditions. Briefly, after PVDF membrane activation, 20 µg of brain cortical homogenates, in a final volume of 5 µL, were placed in dots in specific zones of the membrane. As soon as the dots were dried, the non-specific binding reactions were blocked using 10% BSA for 1 h at room temperature. Thereafter, membranes were incubated with the respective primary antibody overnight at 4 °C. Subsequently, membranes were washed 3 times with TBS-T and incubated with the secondary antibody for 1 h at room temperature. Then, after 3 washes with TBS-T, membranes were incubated with ECF and protein dots visualized using the VersaDoc Imaging System (Bio-Rad, Hercules, CA, USA) and the density of protein dots calculated using the Quantity OneProgram. The antibodies used are listed in Table 3.

### 4.11. Statistical Analysis

Data regarding mitochondrial bioenergetic parameters are presented as mean ± SEM of the indicated number of animals. All other data are expressed as median ± interquartile range. Statistical significance was determined using the one-way ANOVA test for multiple comparisons, followed by the posthoc Tukey–Kramer test with the program GraphPad Prism 6 (GraphPad Software, San Diego, CA, USA). Statistical significance was noted at *p* < 0.05.

## Figures and Tables

**Table 1:** Effect of IHC on brain cortical oxidative phosphorylation system and mitochondrial respiratory function in the 3×Tg-AD mouse model.

		WT	WT IHC	3×Tg-AD	3×Tg-AD IHC
Oxidative Phosphorylation System	ΔΨ (−mV)	220.1 ± 3.16	222.3 ± 5.14	204.6 ± 3.73	221.4 ± 4.65 ^#^
	Repolarization level (−mV)	217.0 ± 2.26	223.7 ± 4.66	205.7 ± 2.7	223.6 ± 4.57 ^##^
	Repolarization lag phase (min)	0.73 ± 0.047	0.69 ± 0.042	0.85 ± 0.086	0.67 ± 0.045
Mitochondrial respiratory function	State 2 (nAtgO/min/mg)	39.09 ± 2.31	35.22 ± 2.12	28.46 ± 2.56	43.44 ± 5.47 ^#^
	RCR	3.80 ± 0.14	3.69 ± 0.36	3.26 ± 0.09 *	3.55 ± 0.17
	ADP/O (nmol ADP/ AtgO/min/mg)	3.94 ± 0.35	4.06 ± 0.36	4.04 ± 0.48	4.03 ± 0.34
	Oligomycin-inhibited respiration (nAtgO/min/mg)	32.49 ± 2.32	40.77 ± 8.37	25.32 ± 1.91	36.20 ± 4.67
	FCCP-stimulated respiration (nAtgO/min/mg))	101.2 ± 11.02	114.0± 23.75	69.78 ± 5.82 *	119.9 ± 16.75

Mitochondrial parameters were evaluated in freshly isolated brain cortical mitochondrial fractions (0.8 mg) in 1 mL of the reaction medium energized with 5 mM succinate (substrate for complex II) in the presence of 2 μM rotenone. Data are the mean ± SEM of 5–7 animals from each experimental condition studied. Statistical significance: * *p* < 0.05 when compared with control WT mice; ^#^ *p* < 0.05, ^##^ *p* < 0.01 when compared with 3×Tg-AD mice. Abbreviations: ΔΨm—mitochondrial membrane potential; RCR—respiratory control ratio.

**Table 2:** Oligonucleotides and Cycling Conditions for qPCR Amplification of ND1 and β-2MG.

Gene	Sequence (5′–3′)	AT (°C)	Amplificon Size (bp)	C
ND1	Sense: GAG CCC TAC GAG CCG TTG CC	58	271	30
	Antisense: GCG AATG GTC CTG CGG CGT A			
β-2MG	Sense: GCG TGG GAG GAG CAT CAG GG	58	264	30
	Antisense: CTC ATC ACC ACC CCG GGG ACT			

Abbreviations: AT—annealing temperature; C—Number of cycles of amplification.

**Table 3:** List of primary and secondary antibodies.

Antibody	Catalog Number	Supplier	Host Specie	Dilution
Aβ (6E10)	SIG-39300	Covance	Mouse	1:1000
DRP1	611113	BD Biosciences	Mouse	1:1000
Dynein	sc-13524	Santa Cruz Biotechnology	Mouse	1:500
Fis1	IMG-5113A	ImGenex	Rabbit	1:750
KIF-5B	Ab5629	Abcam	Rabbit	1:1000
LAMP-1	3243	Cell Signaling	Rabbit	1:500
LC3B (D11)	3868	Cell Signaling	Rabbit	1:1000
Mnf-1	sc-50330	Santa Cruz Biotechnology	Rabbit	1:1000
Mnf-2	sc-100560	Santa Cruz Biotechnology	Mouse	1:1000
OPA-1	612607	BD Biosciences	Mouse	1:1000
Parkin	2132	Cell Signaling	Rabbit	1:1000
PINK-1	sc-517353	Santa Cruz Biotechnology	Mouse	1:500
PSD-95	D27E11	Cell Signaling	Rabbit	1:1000
p^Ser616^-DRP1	3455	Cell Signaling	Rabbit	1:1000
SNAP-25	S5187	Sigma Aldrich	Mouse	1:1000
Synaptotagmin-I	sc-136480	Santa Cruz Biotechnology	Mouse	1:1000
Syntaphilin	sc-365606	Santa Cruz Biotechnology	Mouse	1:500
Tau (AT8)	Mn1020	Thermo Scientific	Mouse	1:750
TFAM	sc-30965	Santa Cruz Biotechnology	Goat	1:750
TOM20	sc-11415	Santa Cruz Biotechnology	Rabbit	1:1000
β-Actin	A5441	Sigma Aldrich	Mouse	1:5000
Mouse IgG alkaline phosphatase conjugate	NIF1316	Amersham Pharmacia Biotech	Goat	1:10,000
Rabbit IgG alkaline phosphatase conjugate	NIF1317	Amersham Pharmacia Biotech	Goat	1:10,000

## Data Availability

The data presented in this study are available in the published article.
